# Acute Kidney Injury Biomarkers for Patients in a Coronary Care Unit: A Prospective Cohort Study

**DOI:** 10.1371/journal.pone.0032328

**Published:** 2012-02-22

**Authors:** Tien-Hsing Chen, Chih-Hsiang Chang, Chan-Yu Lin, Chang-Chyi Jenq, Ming-Yang Chang, Ya-Chung Tian, Cheng-Chieh Hung, Ji-Tseng Fang, Chih-Wei Yang, Ming-Shien Wen, Fun-Chung Lin, Yung-Chang Chen

**Affiliations:** 1 Second Section of Cardiology, Chang Gung Memorial Hospital, Taipei, Taiwan; 2 Department of Nephrology, Chang Gung Memorial Hospital, Taipei, Taiwan; 3 Chang Gung University College of Medicine, Taipei, Taiwan; University of Sao Paulo Medical School, Brazil

## Abstract

**Background:**

Renal dysfunction is an established predictor of all-cause mortality in intensive care units. This study analyzed the outcomes of coronary care unit (CCU) patients and evaluated several biomarkers of acute kidney injury (AKI), including neutrophil gelatinase-associated lipocalin (NGAL), interleukin-18 (IL-18) and cystatin C (CysC) on the first day of CCU admission.

**Methodology/Principal Findings:**

Serum and urinary samples collected from 150 patients in the coronary care unit of a tertiary care university hospital between September 2009 and August 2010 were tested for NGAL, IL-18 and CysC. Prospective demographic, clinical and laboratory data were evaluated as predictors of survival in this patient group. The most common cause of CCU admission was acute myocardial infarction (80%). According to Acute Kidney Injury Network criteria, 28.7% (43/150) of CCU patients had AKI of varying severity. Cumulative survival rates at 6-month follow-up following hospital discharge differed significantly (*p*<0.05) between patients with AKI versus those without AKI. For predicting AKI, serum CysC displayed an excellent areas under the receiver operating characteristic curve (AUROC) (0.895±0.031, *p*<0.001). The overall 180-day survival rate was 88.7% (133/150). Multiple Cox logistic regression hazard analysis revealed that urinary NGAL, serum IL-18, Acute Physiology, Age and Chronic Health Evaluation II (APACHE II) and sodium on CCU admission day one were independent risk factors for 6-month mortality. In terms of 6-month mortality, urinary NGAL had the best discriminatory power, the best Youden index, and the highest overall correctness of prediction.

**Conclusions:**

Our data showed that serum CysC has the best discriminative power for predicting AKI in CCU patients. However, urinary NGAL and serum IL-18 are associated with short-term mortality in these critically ill patients.

## Introduction

Acute kidney injury (AKI) that develops after admission to coronary care unit (CCU) is associated with very poor outcomes [1], [2]. Contrast-induced acute kidney injury (CI-AKI) is an important health issue, as it commonly has a major impact on outcome [3]. The CI-AKI, defined as a ≥25% increase in SCr level, may have an incidence as high as 15% in patients undergoing coronary angiography [4].

Serum creatinine (SCr), however, is a poor marker of early renal dysfunction because serum concentration is greatly influenced by changes in muscle mass and tubular secretion [5], [6]. Hence, the normal reference interval is relatively wide, and SCr alone cannot precisely indicate disease progression. The numerous non-renal factors affecting SCr concentration include body weight, race, age, gender, total body volume, drugs, muscle metabolism and protein intake. Moreover, SCr increases slowly after onset of AKI. By the time a SCr change is noted, a critical therapeutic window may have been missed, particularly in patients with acute tubular necrosis. A troponin-like biomarker of AKI that is easily measured, independent of other biological variables, and that provides both early detection and risk stratification would be a tremendous advance in clinical medicine. The tools of modern science have provided promising AKI biomarkers with potentially high sensitivity and specificity for predicting of AKI and mortality risk after AKI [7]. The aim of this study was to identify the relationship between AKI/short-term prognosis and AKI biomarkers in CCU patients including serum and urinary cystatin C (CysC), neutrophil gelatinase-associated lipocalin (NGAL) and interleukin-18 (IL-18).

## Materials and Methods

### Study design and patient population

This investigation was performed in the CCU at a tertiary care referral center in Taiwan between September 2009 and August 2010. The institutional review board at Chang Gung Memorial Hospital approved the study protocol. All patients gave informed and written consent to participate in the study. In total, 150 critical ill patients were enrolled. Exclusion criteria were as follows: unable to give written informed consent (14 patients), pediatric patient (ages ≤18 years) (3 patients); history of end-stage renal failure (5patients); duration of hospital stay <24 h (10 patients). Readmitted patients were also excluded (6 patients).

### Data collection

Daily measurements of urine output (UO) and SCr began on day 1 of CCU admission and continued for 2 days. Quantification of CysC, NGAL, and IL-18 was performed in serum and urinary samples from all patients on CCU admission. Prospectively collected data were the following: demographic characteristics, reason for CCU admission, primary diagnosis, routine chemistry tests, duration of hospitalization, and outcome. To determine the predictive value of biomarkers at AKI diagnosis, the primary study outcome was AKI onset. To assess the prognostic utility of biomarkers, the secondary outcome was 6-month mortality. After hospital discharge, 6-month follow up examinations were performed *via* records review or telephone interview as needed.

### Definitions

Acute myocardial infarction (AMI) was defined according to the 2007 Expert Consensus Document of Circulation, European Heart Journal [8]. Diagnosis of heart failure was based on Framingham criteria [9]. Sepsis and respiratory failure were defined according to the American College of Chest Physicians/Society of Critical Care Medicine (ACCP/SCCM) Consensus Conference [10]. Illness severity was assessed using Acute Physiology, Age and Chronic Health Evaluation II (APACHE II), which was calculated as described elsewhere [11]. Acute kidney injury was defined as in the Acute Kidney Injury Network (AKIN) classification system, which requires at least two SCr values within 48 h [12]. The classification system comprises individual criteria for SCr levels and UO. A patient can fulfill the criteria via changes in SCr concentrations, changes in UO, or both. Baseline SCr concentration used for AKIN classification was that at the time of CCU admission. The estimated glomerular filtration rate (eGFR) was estimated using the Chinese Modification of Diet in Renal Disease equation [13].

### Sampling and quantifying serum and urinary biomarkers

Blood and urinary samples collected in nonheparinized tubes immediately after CCU admission were centrifuged at 1,500 rpm for 5 minutes. These samples were then stored at −80°C until assay. Serum and urinary CysC and NGAL were measured in duplicate by single ELISA (R&D Systems, Minneapolis, MN, USA). Serum and urinary IL-18 were measured in duplicate by single ELISA (Medical and Biologic Laboratories, Nagoya, Japan) according to manufacturer instructions. Plasma B-type natriuretic peptide (BNP) level was measured by commercial immunodimetric assay method. High-sensitive C-reactive protein (hs-CRP) was measured by autoanalyzer.

### Statistical analysis

Continuous variables were summarized by mean and standard error unless otherwise stated. The primary analysis (the primary end point) was the comparison between AKI and non-AKI groups. Kolmogorov-Smirnov test was used to determine the normal distribution for each variable. Student *t*-test was used to compare the means of continuous variables and normally distributed data; otherwise, Mann-Whitney *U* test was used. Categorical data were tested using the Chi-square test or Fisher exact test. This study utilized the χ^2^ test for trends to assess categorical data associated with AKIN classification. Furthermore, parameters for AKI prediction were assessed using univariate analysis, and variables that were statistically significant (*p*<0.05) in the univariate analysis were included in multivariate analysis by adopting a multiple logistic regression based on forward elimination of data. Risk factors for 6-month mortality (the second end point) were assessed by univariate Cox logistic regression hazard analysis, and statistically significant (*p*<0.05) variables identified by univariate hazard analysis were included in the multivariate analysis by applying multiple logistic forward Cox regression analysis to obtain independent predictors of 6-month survival.

Hosmer-Lemeshow goodness-of-fit test was used for calibration when evaluating the number of observed and predicted deaths in risk groups for the entire range of death probabilities. Discrimination was assessed using the area under a receiver operating characteristic curve (AUROC), which was compared by a nonparametric approach [14]. The AUROC analysis calculated cutoff values, sensitivity, specificity, and overall correctness. Finally, cutoff points were calculated by acquiring the best Youden index [15]. The index is defined as sensitivity+specificity −1, where sensitivity and specificity are calculated as proportions. Youden index has minimum and maximum values of −1 and +1, respectively, with a value of +1 representing the optimal value for an algorithm. Cumulative survival curves as a function of time were generated by Kaplan-Meier approach and compared by log rank test. All statistical tests were two-tailed; a value of *p*<0.05 was considered statistically significant.

## Results

### Subject characteristics

Between September 2009 and August 2010, 150 CCU patients with AMI, congestive heart failure, respiratory failure, or arrhythmias were enrolled. Median subject age was 69-year-old; one hundred and thirteen (75.3%) were male, and thirty-seven (24.7%) were female. The causes of CCU admission were AMI in 120 (80%) patients, congestive heart failure in 21 (14%), respiratory failure in five (3.3%), tachyarrhythmia or bradyarrhythmia in three (2%), and AKI in one patient (0.7%), who was managed conservatively with intravenous inotropic agents plus diuretics and hemodynamic monitoring. No patient required hemodialysis on day 1 of CCU admission.

### AKI and biomarkers

**Figure 1:**
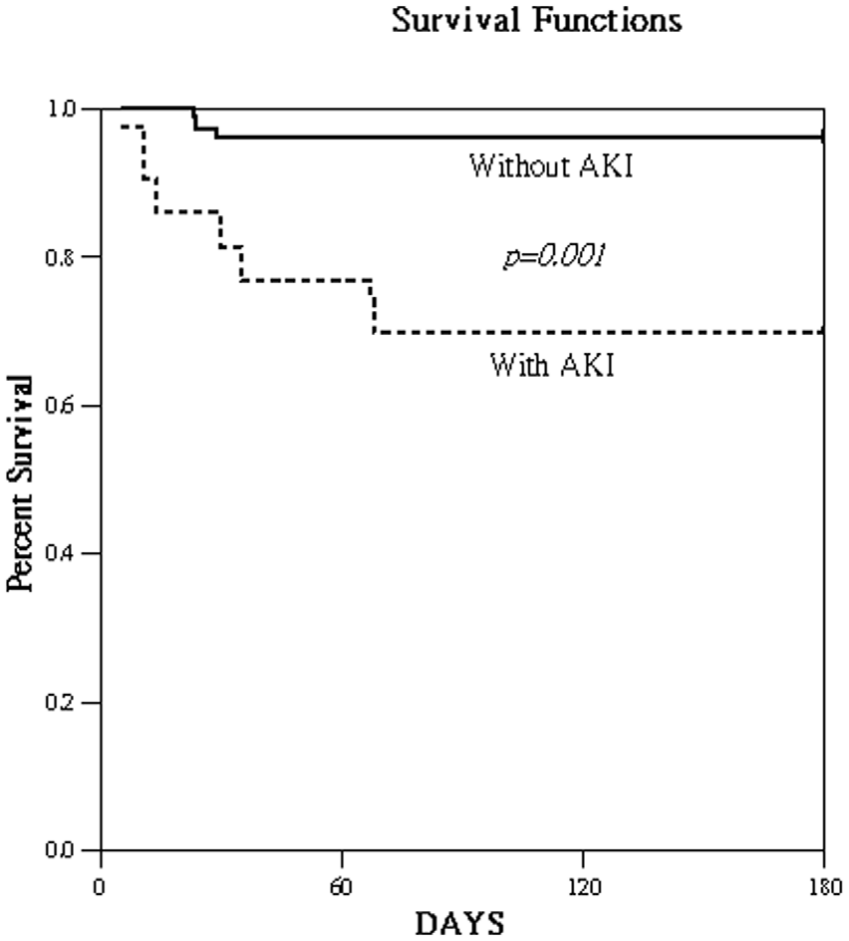
Cumulative survival rate for 150 critically ill patients based on with/without acute kidney injury (AKI) at 48-hour after coronary care unit admission.

Overall, 28.7% (43/150) patients had AKI (AKIN SCr criteria only: 25 patients, UO criteria only: 3 patients, and SCr plus UO criteria: 15 patients). In AKIN classification, 6-month mortality was 3.7% (4/107) for stage-0, 23.5% (4/17) for stage-1, 18.2% (2/11) for stage-2, and 46.7% (7/15) for stage-3 patients (χ2 for trend, *p*<0.001). Table 1 lists that the AKI patients had a significantly high incidence of sepsis, older age as well as high values for APACHE II, blood sugar, baseline SCr concentration, BNP, hs-CRP, CysC, IL-18, and serum and urinary NGAL. They also tended to have low ejection fraction, hemoglobulin (Hb) and serum albumin on CCU day 1 and elevated 6-month mortality (Fig. 1).

**Table 1:** Patient demographic data and clinical characteristics on the first of CCU admission according to with/without AKI.

	All Patients(n = 150)	Without AKI(n = 107)	With AKI(n = 43)	*p*-value
Age (years)	66±1	63±1	73±2	<0.001
Gender, Male (%)	113 (75)	83 (78)	30 (70)	NS (0.316)
Diabetes mellitus, *n* (%)	92 (61)	61 (57)	31 (72)	NS (0.086)
Hypertension, *n* (%)	110 (73)	77 (72)	33 (77)	NS (0.549)
Sepsis, *n* (%)	30 (20)	11 (10)	19 (44)	<0.001
Contrast medium exposure, *n* (%)	114 (76)	84 (79)	30 (70)	NS (0.257)
eGFR less than 60 min/ml, *n* (%)	37 (25)	20 (19)	17 (40)	0.007
Vasoactive/inotropic agents, *n* (%)	30 (20)	19 (18)	11 (26)	NS (0.279)
Mechanical ventilation, *n* (%)	27 (18)	12 (11)	15 (35)	0.001
History of CAD, *n* (%)	110 (73)	82 (77)	28 (65)	NS (0.149)
Mean arterial pressure (mmHg)	80±1	79±2	81±3	NS (0.453)
AST (units/L)	80±13	83±18	72±10	NS (0.711)
Blood sugar (mg/dL)	169±7	158±7	196±13	NS (0.054)
Serum Creatinine (mg/dL)	1.33±0.07	1.19±0.06	1.68±0.27	0.013
Total Bilirubin (mg/dL)	0.6±0.0	0.6±0.0	0.5±0.0	0.042
Albumin (g/L)	3.7±0.0	3.9±0.0	3.4±0.1	<0.001
Hemoglobin (g/dL)	12.7±0.2	13.2±0.2	11.4±0.4	<0.001
Leukocytes (×10^3^/µL)	10.1±0.3	10.0±0.4	10.5±0.6	NS (0.469)
Serum Sodium (mmol/L)	139±0	139±0	139±1	NS (0.958)
hs-CRP (mg/L)	42±5	34±6	62±10	0.002
Troponin I (ng/mL)	6.8±1.1	4.6±0.9	12.9±3.4	0.022
BNP (pg/mL)	690±73	458±65	1268±169	<0.001
Ejection fraction (%)	51±1	53±1	45±3	0.011
APACHE II (points)	10±1	8±1	14±1	<0.001
Serum NGAL (ng/mL)	149±11	110±8	259±29	<0.001
Urine NGAL (ng/mL)	74±17	22±4	221±60	0.002
Serum IL-18 (pg/mL)	450±21	397±25	582±31	<0.001
Urine IL-18 (pg/mL)	71±5	60±4	98±16	0.026
Serum CysC (mg/L)	2.1±0.2	1.2±0.1	4.1±0.7	<0.001
Urine CysC (mg/L)	2.9±1.3	1.3±0.4	7.0±4.4	NS (0.204)

AKI, acute kidney injury; APACHE, Acute Physiology and Chronic Health Evaluation; AST, aspartate aminotransferase; BNP, B-type natriuretic peptide; CAD, coronary artery disease; CCU, coronary care unit; CysC, cystatin C; eGFR, estimated glomerular filtration rate; hs-CRP, high-sensitivity C reactive protein; IL-18, interleukin-18; NGAL: neutrophil gelatinase-associated lipocalin; NS, not significant.

To assess calibration, Table 2 shows the goodness-of-fit indices as measured by the Hosmer-Lemeshow chi-square analysis of predicted AKI risk and the predictive accuracy of the APACHE II, serum and urinary CysC, NGAL and IL-18 for these patients. Table 2 also compares the discriminatory power of APACHE II and these AKI biomarkers. The AUROC analysis confirmed that serum CysC was the best AKI predictor in the overall CCU population (AUROC 0.895±0.031, *p*<0.001). There were no significant changes in AUROCs for urinary NGAL, IL-18 or CysC after normalizing for urine creatinine (Ucr, data not shown).

**Table 2:** Comparison of calibration and discrimination of the biomarkers and APACHE II on the first of CCU admission in predicting AKI and 6-month mortality.

	Calibration			Discrimination		
	goodness-of-fit (χ^2^)	df	*p*	AUROC ± SE	95% CI	*p*
*For AKI prediction*						
Serum NGAL	10.591	8	0.226	0.828±0.036	0.758–0.898	<0.001
Urine NGAL	13.602	8	0.093	0.796±0.044	0.710–0.882	<0.001
Serum IL-18	23.873	8	0.002	0.773±0.038	0.698–0.847	<0.001
Urine IL-18	13.008	8	0.112	0.621±0.060*	0.504–0.738	0.032
Serum CysC	9.491	8	0.303	0.895±0.031	0.834–0.956	<0.001
Urine CysC	8.793	8	0.360	0.633±0.061*	0.514–0.751	0.019
APACHE II	10.717	8	0.151	0.757±0.039	0.690–0.844	<0.001
*For 6-month mortality prediction*						
Serum NGAL	12.185	8	0.143	0.836±0.049	0.740–0.933	<0.001
Urine NGAL	10.701	8	0.219	0.886±0.036	0.815–0.957	<0.001
Serum IL-18	13.829	8	0.086	0.841±0.055	0.734–0.948	<0.001
Urine IL-18	11.702	8	0.165	0.755±0.077	0.604–0.906	0.001
Serum CysC	5.439	8	0.710	0.861±0.044	0.774–0.948	<0.001
Urine CysC	5.577	8	0.695	0.567±0.089†	0.391–0.742	0.375
APACHE II	4.347	8	0.825	0.858±0.061	0.764–0.952	<0.001

AKI, acute kidney injury; APACHE, Acute Physiology and Chronic Health Evaluation; AUROC, areas under the receiver operating characteristic curve; CI, confidence intervals; CysC, cystatin C; df, degree of freedom; IL-18, interleukin-18; NGAL: neutrophil gelatinase-associated lipocalin; SE, standard error.

* , *p*<0.05 vs. Serum NGAL and CysC;

† , *p*<0.05 vs. APACHE II, Urine NGAL and Serum CysC.

Univariate analysis identified 15 of the 30 variables (Table 1) as AKI prediction valuable (Table 3). Multivariate analysis identified the following variables as of independent predicting AKI significance: serum NGAL and CysC (Table 3). Regression coefficients of these variables were utilized to calculate a logit of death for each patient as follows:
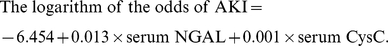



**Table 3:** Logistic regression analysis for AKI, according to baseline prognostic factors on the first day of CCU admission.

Parameter	BetaCoefficient	Standard error	Odds ratio(95%CI)	*p*
*Univariate logistic regression*				
Age	0.085	0.020	1.089 (1.048–1.132)	<0.001
Sepsis	1.933	0.442	6.909 (2.904–16.440)	<0.001
eGFR less than 60 min/ml	1.045	0.398	2.844 (1.303–6.211)	0.009
Mechanical ventilation	1.445	0.443	4.241 (1.780–10.105)	0.001
Serum Creatinine, CCU first day	0.548	0.196	1.729 (1.177–2.540)	0.005
Albumin	−2.382	0.485	0.092 (0.036–0.239)	<0.001
Hemoglobin	−0.345	0.087	0.708 (0.597–0.840)	<0.001
BNP	0.001	0.000	1.001 (1.001–1.002)	<0.001
Ejection fraction	−0.034	0.012	0.967 (0.945–0.989)	0.004
APACHE II	0.158	0.036	1.171 (1.092–1.256)	<0.001
Serum NGAL	0.009	0.002	1.009 (1.005–1.013)	<0.001
Urine NGAL	0.012	0.004	1.012 (1.004–1.020)	0.004
Serum IL-18	0.003	0.001	1.003 (1.001–1.004)	<0.001
Urine IL-18	0.013	0.005	1.013 (1.004–1.022)	0.007
Serum CysC	0.002	0.000	1.002 (1.001–1.002)	<0.001
*Multivariate logistic regression*				
Serum NGAL	0.013	0.006	1.013 (1.001–1.025)	0.028
Serum CysC	0.001	0.001	1.001 (1.000–1.003)	0.036
constant	−6.454	1.184	-	-

AKI, acute kidney injury; APACHE, Acute Physiology and Chronic Health Evaluation; BNP, B-type natriuretic peptide; CCU, coronary care unit; CI, confidence intervals; CysC, cystatin C; eGFR, estimated glomerular filtration rate; IL-18, interleukin-18; NGAL: neutrophil gelatinase-associated lipocalin.

### Short-term mortality and AKI biomarkers

The overall 6-month mortality rate was 11.3% (17/150). Table 4 lists patient demographic data and clinical characteristics for both 6-month survivors and non-survivors. Of thirty variables (Table 4), univariate Cox logistic regression hazard analysis identified 15 with prognostic value for 6-month mortality (Table 5). Multiple Cox logistic regression hazard analysis revealed that APACHE II, serum sodium, urinary NGAL and serum IL-18 on CCU admission day one were independent risk factors for 6-month mortality (Table 5).

**Table 4:** Patient demographic data and clinical characteristics on the first of CCU admission according to 6-month mortality.

	Survivors(n = 133)	Non-survivors(n = 17)	*p*-value
Age (years)	64±1	75±2	<0.001
Gender, Male (%)	103 (77)	10 (59)	NS (0.094)
Diabetes mellitus, *n* (%)	84 (63)	8 (47)	NS (0.199)
Hypertension, *n* (%)	95 (71)	15 (88)	NS (0.140)
Sepsis, *n* (%)	22 (17)	8 (47)	0.003
Contrast medium exposure, *n* (%)	104 (78)	10 (59)	NS (0.078)
eGFR less than 60 min/ml, *n*(%)	27 (20)	10 (59)	0.001
Vasoactive/inotropic agents, *n* (%)	28 (21)	2 (12)	NS (0.367)
Mechanical ventilation, *n* (%)	18 (14)	9 (53)	<0.001
History of CAD, *n* (%)	101 (76)	9 (53)	0.043
Mean arterial pressure (mmHg)	79±2	84±5	NS (0.307)
AST (units/L)	76±14	106±21	NS (0.479)
Blood sugar (mg/dL)	171±7	158±9	NS (0.314)
Serum Creatinine (mg/dL)	1.25± 0.07	1.97±0.30	0.031
Total Bilirubin (mg/dL)	0.6±0.0	0.7±0.1	NS (0.297)
Albumin (g/L)	3.8±0.0	3.2±0.1	<0.001
Hemoglobin (g/dL)	13.0±0.2	10.6±0.4	<0.001
Leukocytes (×10^3^/µL)	9.8±0.3	12.5±1.1	0.007
Serum Sodium (mmol/L)	139±0	136±1	0.005
hs-CRP (mg/L)	32±4	104±30	0.030
Troponin I (ng/mL)	5.5±1.0	17.1±5.8	NS (0.068)
BNP (pg/mL)	560±67	1714±281	0.001
Ejection fraction (%)	52±1	42±5	NS (0.073)
APACHE II (points)	9±0	19±2	<0.001
Serum NGAL (ng/mL)	126±9	320±51	0.002
Urine NGAL (ng/mL)	36±8	351±114	0.014
Serum IL-18 (pg/mL)	411±18	747±91	0.002
Urine IL-18 (pg/mL)	62±3	141±38	NS (0.055)
Serum CysC (mg/L)	1.6±0.1	5.6±1.5	0.021
Urine CystC (mg/L)	1.2±0.4	16.4±10.6	NS (0.173)

APACHE, Acute Physiology and Chronic Health Evaluation; AST, aspartate aminotransferase; BNP, B-type natriuretic peptide; CAD, coronary artery disease; CCU, coronary care unit; CysC, cystatin C; eGFR, estimated glomerular filtration rate; hs-CRP, high-sensitivity C reactive protein; IL-18, interleukin-18; NGAL: neutrophil gelatinase-associated lipocalin; NS, not significant.

**Table 5:** Cox regression analysis for all-cause 6-month mortality, according to baseline prognostic factors on the first day of CCU admission.

Parameter	BetaCoefficient	Standard error	Hazard ratios(95%CI)	*p*
*Univariate hazard analysis*				
Age (years)	0.072	0.025	1.075 (1.023–1.129)	0.004
Mechanical ventilation	1.778	13.355	5.919 (2.281–15.362)	<0.001
Sepsis	1.393	0.486	4.026 (1.553–10.441)	0.004
eGFR less than 60 min/ml	1.724	0.538	5.608 (1.954–16.905)	0.001
Serum Creatinine, CCU first day	0.519	0.178	1.680 (1.186–2.382)	0.004
Albumin	−1.933	0.450	0.145 (0.060–0.350)	<0.001
Hemoglobin	−0.401	0.105	0.669 (0.545–0.823)	<0.001
Serum Sodium	−0.199	0.064	0.819 (0.723–0.928)	0.002
hs-CRP	0.011	0.003	1.011 (1.006–1.016)	<0.001
BNP	0.001	0.000	1.001 (1.000–1.001)	<0.001
APACE II	0.160	0.027	1.173 (1.113–1.237)	<0.001
Serum NGAL	0.007	0.001	1.007 (1.005–1.010)	<0.001
Urine NGAL	0.003	0.000	1.003 (1.002–1.004)	<0.001
Serum IL-18	0.003	0.001	1.003 (1.002–1.004)	<0.001
Urine IL-18	0.005	0.001	1.005 (1.003–1.007)	<0.001
*Multivariate hazard analysis*				
Urine NGAL	0.003	0.001	1.003 (1.001–1.004)	<0.001
Serum IL-18	0.005	0.001	1.005 (1.003–1.006)	<0.001
APACE II	0.129	0.036	1.138 (1.061–1.221)	<0.001
Serum Sodium	−0.352	0.103	0.703 (0.575–0.860)	0.001

APACHE, Acute Physiology and Chronic Health Evaluation; BNP, B-type natriuretic peptide; CCU, coronary care unit; CI, confidence intervals; hs-CRP, high-sensitivity C reactive protein; IL-18, interleukin-18; NGAL: neutrophil gelatinase-associated lipocalin.

Table 2 shows the goodness-of-fit, as measured by the Hosmer-Lemeshow chi-square statistic of predicted 6-month mortality risk, for the predictive accuracy of the APACHE II and AKI biomarkers. Table 2 also compares discriminatory power for APACHE II, serum and urinary CysC, NGAL, and IL-18. The AUROC analysis verified that urinary NGAL had the best discriminatory power for predicting 180-day mortality. To assess the predictive value of selected cut-offs for predicting 6-month mortality, the sensitivity, specificity and overall correctness of prediction were determined. Table 6 summarizes the data calculated using the cutoff point providing the best Youden index. The urinary NGAL had the best Youden index and highest overall correctness of prediction.

**Table 6:** Predictions of the biomarkers and APACHE II on the first of CCU admission in predicting AKI and 6-month mortality.

PredictiveFactors	CutoffPoint	Youden Index	Sensitivity (%)	Specificity (%)	Overall Correctness (%)
*For AKI prediction*					
Serum NGAL	110*	0.61	92	69	81
Urine NGAL	33*	0.50	66	84	75
Serum IL-18	374*	0.62	97	65	81
Urine IL-18	70*	0.34	50	84	67
Serum CysC	1.8*	0.68	77	91	84
Urine CysC	0.2*	0.30	46	84	65
APACHE II	7*	0.39	93	46	70
*For 6-month mortality prediction*					
Serum NGAL	135*	0.64	88	76	82
Urine NGAL	33*	0.68	88	80	84
Serum IL-18	442*	0.65	94	71	83
Urine IL-18	77*	0.43	67	76	72
Serum CysC	2.1*	0.67	77	90	84
Urine CysC	0.2*	0.27	47	80	64
APACHE II	14*	0.64	77	87	82

AKI, acute kidney injury; APACHE, Acute Physiology and Chronic Health Evaluation; CysC, cystatin C; IL-18, interleukin-18; NGAL: neutrophil gelatinase-associated lipocalin.

* Value giving the best Youden index (sensitivity+specificity −1).

## Discussion

This study confirmed that serum CysC, IL-18, NGAL, and urinary NGAL on CCU admission day one were prognostically significant biomarkers of AKI. This study also confirmed that APACHE II, serum sodium, urinary NGAL and serum IL-18 on CCU admission day 1 were strongly correlated with 180-day mortality.

McCullough and colleagues reported that renal risk stratification can identify groups with high rates of CCU death and poor long-term survival. This risk is only partially explained by comorbidities, including diabetes, age and congestive heart failure (CHF) [16]. This investigation found that 21 CHF patients as the mean reason for CCU admission had higher 6-month mortality rate (28.6%). Increased SCr, hyperbilirubinemia, and dilutional hyponatremia are observed in severe CHF cases. Dilutional hyponatremia occurs in patients with decreased free-water clearance driven by nonosmotic secretion of vasopressin secondary to circulatory dysfunction and effective hypovolemia [17]. Linear correlation analysis revealed a converse correlation between serum sodium concentration and SCr levels on the first day of CCU admission (r = −0.237, *p* = 0.004) in this study.

Normally, CysC, which is a 13-kDa protein, is filtered freely and completely reabsorbed and catabolized within the proximal tubule. Some limitations of the SCr level, including the effects of gender, body muscle mass, and diet, did not significantly influence serum CysC levels. It is a relatively stable protein in serum that shows promise as a convenient measure of glomerular filtration rate (GFR) [18]. Moreover, although CysC is normally not detected in urine, elevated urine CysC levels may indicate tubular epithelial damage, and has been proposed as an additional urine biomarker for AKI [19]. Unlike other potential AKI biomarkers, CysC is not affected by storage conditions or interfering substances and does not increase with urinary tract infection or other chronic non-kidney disease states, such as malignancy [20]. Because it is a marker of glomerular filtration, the protein is valuable for identifying established AKI. As a predictor of dialysis requirements or in-hospital death in AKI, serum CysC level is as accurate as SCr level, serum urea nitrogen level, and urine output [21]. Furthermore, Heise and colleagues reported that neither urinary CysC concentrations nor their product with the creatinine ratios showed any significant differences between the AKI and non-AKI patient groups [22]. In this study, serum but not urinary CysC level had better discriminatory power, Youden index, and overall accuracy in predicting AKI compared to other biomarkers.

Urinary NGAL concentrations were significantly higher in patients with AKI and accurately discriminated between both groups with satisfactory sensitivity and specificity. However, the variability of urinary NGAL within both groups was much higher than that in an earlier study of pediatric patients by Mishra et al. [23], in which the authors noted several rare comorbidities in young patients, which may increase NGAL excretion independent of kidney injury. Such conditions include chronic heart failure [24], atherosclerosis [25], vascular injury [26], the need for dialysis within the first week of kidney transplantation [27] and neutrophilic inflammation [28]. Despite these confounding factors, the present study shows that urinary NGAL accurately indicates AKI and short-term mortality in adult patients with a broad spectrum of comorbidities.

A large study of healthy, middle-aged European men also showed that serum IL-18 concentration is an independent predictor of coronary events [29]. Additionally, variation within the IL-18 gene is known to influence circulating concentrations of IL-18 and clinical outcome in patients with coronary heart disease (CHD) [30]. The exact influence of IL-18 on CHD risk remains unclear. The IL-18 shares similar downstream signaling pathways with several other cytokines (*e.g.*,interaction with MyD88 [31] and nuclear translocation of NF-κB [32]), but this may not be true for all cell types [33]. Animal studies indicate that both IL-6 and IL-1 induce fever [34] whereas IL-18 does not [35], which suggests substantial differences in site and mode of action between these cytokines. Also, IL-18 reportedly predicts CHD events independent of CRP [29], which further supports this hypothesis. Therefore, if IL-18 participates in an alternative inflammatory pathway in CHD, knowledge of important genetic effects may improve risk prediction beyond that of other inflammatory mediators [36]. Moreover, 6-month mortality rates differed significantly according to the best Youden index below and above the cut-off value of 442 pg/mL serum IL-18 (1.1% *vs.* 27.6%, *p*<0.001) in this investigation.

A recently published article reported the incidence, cost, and outcome of severe sepsis in the United States. Analysis of a large sample from major centers identified an incidence of severe sepsis as 3 cases per 1,000 people, and 2.26 cases per 100 hospital discharges. Out of these cases, 51.1% were admitted to intensive care units; an additional 17.3% were cared for in intermediate care units or CCUs [37]. In this investigation, the incidence of CCU patients with sepsis was roughly 20% (30/150) (Table 1). This high sepsis incidence can be attributed to the cause of CCU admission; that is, a high proportion of patients had congestive heart failure (14%) and respiratory failure (3.3%). Furthermore, the overall CI-AKI in this study was 26.3% (30/114), which is high compared to that in other studies [4]. This high AKI incidence may be attributable to inclusion of 37 patients with an eGFR <60 min/ml and 30 septic patients; both of which are positively associated with AKI.

Despite the promising results obtained in this study, several important limitations are noted. First, this study was conducted at just one institution. Therefore, the results may not be directly extrapolated to other patient populations. Second, predictions vary among individuals; that is, a prediction is only an approximate indicator of mortality risk in specific subjects. Of all CCU patients in this cohort, most had ischemic AKI. Notably, AKI is often multifactorial, and experimental results require validation by a relatively larger randomized prospective trial. Third, the AKI definition was based on elevated SCr concentrations and/or oliguria, which raises the problem of using a flawed “gold standard” to analyze the performance of novel biomarkers [38]. This is an important limitation of this study. Besides, the use of the first measured SCr at the ICU admission can actually decrease the diagnosis of AKI, since some patients will already have AKI at the time of ICU admission. On the other hand, those patients could have higher levels of biomarkers, interfering with the performance of these markers to predict and prognosis AKI. Fourth, the number of mechanically ventilated patients (n = 27), patients with eGFR less than 60 min/ml (n = 37) and one pre-existing AKI patient as well as outcome events was insufficient to determine independent risk factors for AKI and 6-month mortality by using multivariate techniques. Recent evidence suggests that the predictive ability of biomarkers for AKI is reduced in patients with chronic kidney disease [39]. Finally, sequential measurement of these AKI biomarkers (*e.g.*, daily, weekly) or other AKI biomarkers (*e.g.*, kidney injury molecule-1, liver fatty acid-binding proteins) may reflect the dynamic aspects of clinical diseases and thus provide superior information on mortality risk.

The analytical data demonstrated the good discriminative power of serum CysC and NGAL levels for predicting AKI in these critically ill patients. This study also indicated that APACHE II, serum sodium concentrations, urinary NGAL and serum IL-18 on day 1 of CCU admission are independent predictors of 180-day mortality. The AKI biomarkers panel is useful when timing the initial insult and predicting AKI duration, severity and clinical outcomes. However, because of the relatively small sample size, the predictive accuracies of serum CysC, NGAL, and IL-18 levels and urinary NGAL of CCU population require further external validation.
